# Performance evaluation of a motor-imagery-based EEG-Brain computer interface using a combined cue with heterogeneous training data in BCI-Naive subjects

**DOI:** 10.1186/1475-925X-10-91

**Published:** 2011-10-12

**Authors:** Donghag Choi, Yeonsoo Ryu, Youngbum Lee, Myoungho Lee

**Affiliations:** 1 Department of Electrical and Electronic Engineering, Yonsei University, 134 Shinchon-dong, Seodaemun-gu, Seoul, 120-749, Korea

**Keywords:** EEG, Brain Computer Interface, Motor Imagery, Heterogeneous and Homogeneous Combined Cue

## Abstract

**Background:**

The subjects in EEG-Brain computer interface (BCI) system experience difficulties when attempting to obtain the consistent performance of the actual movement by motor imagery alone. It is necessary to find the optimal conditions and stimuli combinations that affect the performance factors of the EEG-BCI system to guarantee equipment safety and trust through the performance evaluation of using motor imagery characteristics that can be utilized in the EEG-BCI testing environment.

**Methods:**

The experiment was carried out with 10 experienced subjects and 32 naive subjects on an EEG-BCI system. There were 3 experiments: The experienced homogeneous experiment, the naive homogeneous experiment and the naive heterogeneous experiment. Each experiment was compared in terms of the six audio-visual cue combinations and consisted of 50 trials. The EEG data was classified using the least square linear classifier in case of the naive subjects through the common spatial pattern filter. The accuracy was calculated using the training and test data set. The *p*-value of the accuracy was obtained through the statistical significance test.

**Results:**

In the case in which a naive subject was trained by a heterogeneous combined cue and tested by a visual cue, the result was not only the highest accuracy (*p *< 0.05) but also stable performance in all experiments.

**Conclusions:**

We propose the use of this measuring methodology of a heterogeneous combined cue for training data and a visual cue for test data by the typical EEG-BCI algorithm on the EEG-BCI system to achieve effectiveness in terms of consistence, stability, cost, time, and resources management without the need for a trial and error process.

## Background

Recently, BCI technology has progressed as state-of-the-art medical devices to control and communicate with applicable accessories such as artificial limbs, prosthetic and wheelchair using the signal of brain activity [1-3]. There have been numerous studies of brainwaves collected electrically from brain activity on the cortex related to the brain computer interface [4-6]. Other research has investigated the types of electrical brain activity that can be used to implement the EEG-BCI systems [7-9].

The electroencephalographic (EEG) mu rhythm is an 8-13 Hz rhythm generated by the sensorimotor cortex that is most prominent when subjects are resting and is attenuated or abolished when subjects move or observe biological movements [10,11]. The μ-rhythm is capable of transforming by itself as opposed to the brain activity events [12]. Motor imagery implies a thought activity of imagining of physical movement. Without such physical activity it can transform the μ-rhythm within the sensory motor cortex [13]. In other words, when imagining a hand movement or actually moving the hand, ERD (Event-Related Desynchronization) occurs around the μ-rhythm area within the sensory motor cortex [14,15].

However, the subjects in EEG-BCI system experience difficulties when attempting to obtain the consistent performance of the actual movement by motor imagery alone [7,16]. Hence, it is necessary to find the condition that affect the performance factors of the EEG-BCI system to guarantee equipment safety and trust through an evaluation of the performance of the EEG-BCI system using motor imagery characteristics that can be utilized in the EEG-BCI testing environment.

In a naive homogeneous training data experiment, Kim found that the types of training data do not affect the level of accuracy [16]. In an homogeneous training data experiment, Lee carried out a BCI experiment with experienced subjects and cross-compared three classifiers of combined cues (i.e., audio-visuals cues), analyzing the levels of statistical significance and cross-correlation [17]. However, Lee's study lacked statistical confidence, as the subjects in the experiment built experience based only on ten trials. Thus, in addition to the sample size limitation, the experiment also lacked any consideration of naive subjects.

In an effort to mitigate the sample size issue and include additional variables designed to improve the concentration of the subjects. Furthermore, while the existing combined cue experiment is based on homogeneous training data, this experiment is based on the heterogeneous data, which provides a useful comparative analysis. It should be noted that for the heterogeneous case, the cue speed is a mix of four- and two-second durations. Hence, the key comparisons made here are the naive homogeneous training data experiment and heterogeneous training data experiment.

## Methods

### Subject and data acquisition

The experiment was carried out with 10 experienced subjects aged 23.9 ± 2.5 and 32 naive subjects aged 23.5 ± 1.8 without encephalopathy, mental health disorder and selfmutilation. There were 3 experiments: The experienced homogeneous experiment, the naive homogeneous experiment and the naive heterogeneous experiment. The characteristics of each experiment are shown in Table 1.

**Table 1:** Characteristics of the experiments

Characteristics of experiment	Experienced homogeneous experiment	Naive homogeneous experiment	Naive heterogeneous experiment
**Experience of subject**	Experienced	Naïve (i.e., no experience)	

**Number of subjects**	10 men/women	32 men/women	

**Average age of subjects**	23.9 ± 2.5	23.5 ± 1.8	

**Stimuli type of training data**	Homogeneous	Homogeneous	Heterogeneous

**Duration of a cue in one trial**	7 seconds	7 seconds	6 or 8 seconds

**Number of trials**	50 trials		

The experiments consists of 3 experiments: The experienced homogeneous experiment using the homogeneous training data with experienced subjects, the naive homogeneous experiment using the homogeneous training data with naive subjects and the naive heterogeneous experiment using the heterogeneous training data with naive subjects.

**Figure 1:**
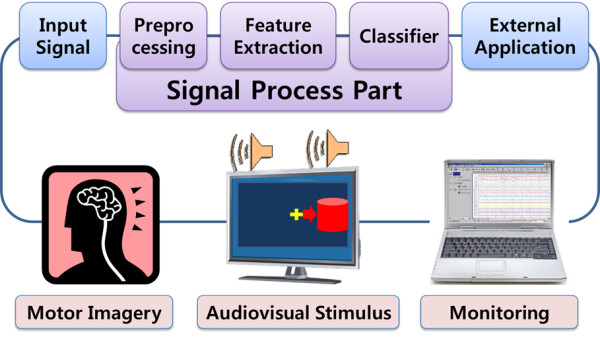
**Block diagram of EEG-BCI system**. In order to make the subject execute the motor imagery tasks, audio-visual stimuli were presented to the subject via a monitor and the speaker of the computer, which were controlled by the experiment manager.

The block diagram of the EEG-BCI system showed the signal acquisition and process in Figure 1. The brain activity signals were extracted at specific locations using a golden disk electrode with a hole. The locations of F3, Fz, F4, FC1, FCz, FC2, C3, C1, Cz, C2, C4, CP1, CP2, P3, Pz, and P4 were selected according to the extended international 10/20 system. The impedance of each electrode was 5 kΩ or less. The EEG acquisition and management system (model: WEG-32, Laxtha Inc. Korea) generated EEG data using an input signal from an amplifier and transferred the EEG data to a notebook computer connected by a USB interface.

The EEG data was classified using the least square (LS) linear classifier in case of the naive subjects through the common spatial pattern (CSP) filter. We calculated the accuracy using the training data set and test data set. The *p*-value of the accuracy was obtained through the statistical significance test (i.e., t-test). In case of the experienced subjects, we used three types of classifiers: the least square (LS) linear classifier using a linear matrix equation and a pseudo inverse matrix, the support vector machine (SVM) using a pattern classifier based on structural risk minimization, and linear discriminant analysis (LDA) using the method reducing the dimension of the data feature vector by maximizing the ratio between the intra-classes-distribution and the inter-classes-distribution. The accuracy as to how the EEG-BCI reading of the brainwave collected matches the actual instructed data is then recorded.

### Experimental procedure

There are six cue-combinations in total, comprised of two visual cues, two auditory cues and two combined cues as shown in Table 2.

**Table 2:** Cue-combinations in the experiment

Cue-combinations	Training	Testing
VA	Visual Cue	Auditory Cue

AV	Auditory Cue	Visual Cue

CA	Combined Cue	Auditory Cue

CV	Combined Cue	Visual Cue

VC	Visual Cue	Combined Cue

AC	Auditory Cue	Combined Cue

There are six cue-combinations as VA, AV, CA, CV, VC, and AC. For example, in the case of the CV combination, the experiment used the combined cue for training and the visual cue for testing.

Each combination consists of 50 trials. For the training data, the time for each cue is 6 or 8 seconds followed by 2 seconds of a blank screen, 2 seconds of a fixation cross and 2 or 4 seconds of cue intervals to train the motor imagery. For the test data, the time for each cue is 7 seconds, and this is followed by 2 seconds of a blank screen, 2 seconds of a fixation cross and 3 seconds of cue intervals to test the motor imagery. The subject imagines the motor imagery according to the audio-visual cue instructions presented on the computer monitor and speaker that are operated by the experiment manager.

Table 3 shows one trial of cue presentations using a cue. If the cue presented only visually, then the cue is referred to as a visual cue. If the cue presented only as an auditory cue, then the cue is referred to as an auditory cue, and if the cue presented as both an auditory and a visual cue, then the cue is considered as a combined cue. The cueing method and presenting duration are randomly distributed, eliminating the possibility of prediction through training.

**Table 3:** One trial of cue presentation

Experi-mental Phase	Display Blank Screen	Display Fixation Cross	Beep for Starting	Sound "Left" or "Right"	Display Red Cylinder on Left or Right	Beep for Stopping
Homo-geneous cue	2 seconds	2 seconds	3 seconds			

Hetero-geneous cue	2 seconds	2 seconds	2 seconds or 4 seconds, randomly			

Stimuli II Screen and/or Sound						
			
						

One experiment consisted of 50 trials. One trial required 7 and 6 or 8 seconds for presenting the cue to each subject for a motor imagery time of 3 and 2 or 4 seconds with audio-visual stimuli presented randomly and unpredictably.

### Experienced homogeneous experiment

The experimental results of the experienced subjects using homogeneous training data derived from a homogeneous stimulus having the same cue time for motor imagery is presented for 3 seconds in case of the training data and 3 seconds in case of the test data, as shown in Table 3. For example, if we used the combined cue for the left motor imagery training tasks, a blank screen was presented to the experienced subject for 2 seconds and the fixation cross was continuously presented for 2 seconds. Finally, a red cylinder on the left side of the monitor with the beep sound and the "left" sound of the speaker were presented for 3 seconds simultaneously for motor imagery.

### Naive homogeneous experiment

The experimental results of the naive subjects using the homogeneous training data derived from a homogeneous stimulus having the same cue time for motor imagery is presented for 3 seconds for training and 3 seconds for testing as shown in Table 3. For example, if we used the visual cue for the right motor imagery training tasks, a blank screen was presented to the experienced subject for 2 seconds and the fixation cross was continuously presented for 2 seconds. Finally, a red cylinder on the right side of the monitor with the beep sound was presented for 3 seconds for motor imagery.

### Naive heterogeneous experiment

The experimental results using the naive heterogeneous training data derived from 2 types of heterogeneous stimuli having a different cue time was presented for 2 or 4 seconds in case of the training data as shown in Table 3. For example, if we used the combined cue for the right motor imagery training tasks, a blank screen was presented to the experienced subject for 2 seconds and the fixation cross was continuously presented for 2 seconds. Finally, a red cylinder on the right side of the monitor with the beep sound and the "right" sound of the speaker were presented for 2 or 4 seconds simultaneously for motor imagery. The duration of the last phase in this trial was randomized.

## Results

### Experienced homogeneous experiment

The experiment with experienced subjects using homogeneous training data sought to investigate the responses to a combination of visual and auditory (i.e., audio-visual) cues. Furthermore, a comparative evaluation was done based on the three classifiers (LS, SVM, and LDA), followed by a statistical analysis to investigate the significance and correlation between the six combinations used in the audio-visual cue experiment.

Subject number 1 had an accuracy score of 0.8 for all three classifiers (LS, SVM, and LDA). The second highest performers were numbers 4 and 8, who scored above 0.6. Subject number 3 had the lowest accuracy mark for all three classifiers (LS, SVM, and LDA).

**Figure 2:**
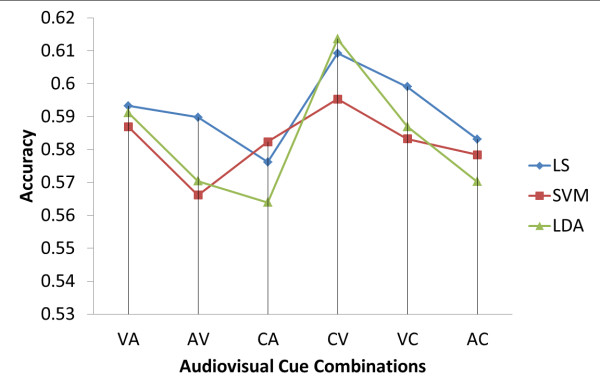
**Result of the experienced homogeneous experiment**. 3 classifiers: The least square (LS), support vector machine (SVM), the linear discriminant analysis (LDA) were compared in terms of six cue combinations.

The plots in Figure 2 show the accuracy of the three classifiers (LS, SVM, and LDA) for all six combinations. It was found that the CV and VC combinations have higher accuracy levels than the others for all three classifiers (LS, SVM, and LDA). The CV accuracy is higher than that of CA (p < 0.05) in the case of LDA, while the other cases show no statistical significance.

### Naive homogeneous experiment

**Figure 3:**
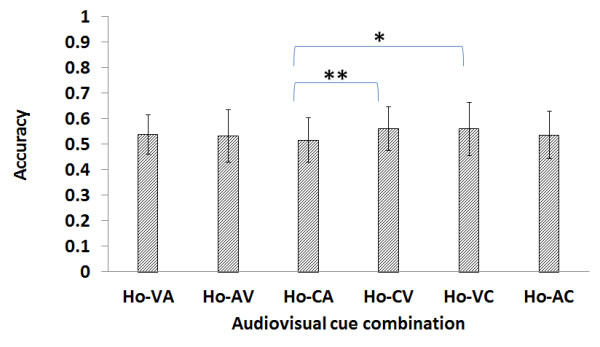
**Result of the naive homogeneous experiment**. The Ho-CV (i.e., homogeneous CV combination) accuracy is higher than the Ho-CA accuracy (p < 0.01). The Ho-VC is also higher than the Ho-CA accuracy (p < 0.05).

Figure 3 shows the result of the experiment using homogeneous training data with the naive subjects in average accuracy of the six cue-combinations. Ho-CV shows higher average accuracy than Ho-CA (*p *< 0.01). Ho-VC shows higher average accuracy than Ho-CA (*p *< 0.05).

**Figure 4:**
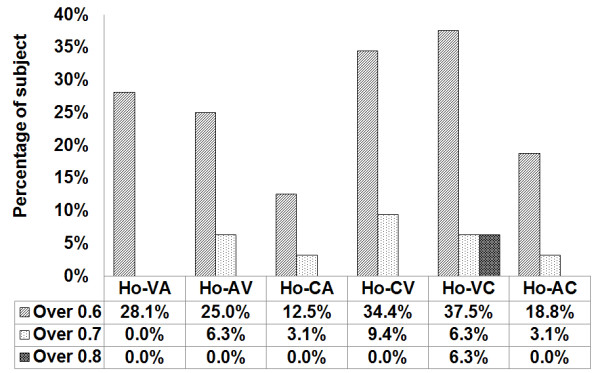
**Result of the naive homogeneous experiment greater than 0.6 in terms of accuracy**. The experiment using homogeneous training data shows the percentages of subject who scored greater than 0.6. Ho-CV and Ho-VC show higher percentage than others. Ho-VC shows higher percentage than Ho-CV.

Figure 4 shows the results of the analyses of subjects whose average accuracy levels are greater than 0.6, 0.7 and 0.8 in terms of accuracy according to the cue combination of the Ho-CV combination of a combined cue for training and a visual cue for testing, and the Ho-VC combination of a visual cue for training and a combined cue for testing. Ho-CV and Ho-VC show higher percentage than others. Ho-VC shows higher percentage than Ho-CV.

The analysis criteria are based on the six combinations described in Table 1. The results shown in Figure 3 are more general considering that they only show the general tendency in terms of the average. In comparison, Figure 4 is more indicative of the actual performance capacity, as the experiment was limited to subjects who scored above a certain level of accuracy.

### Naive heterogeneous experiment

**Figure 5:**
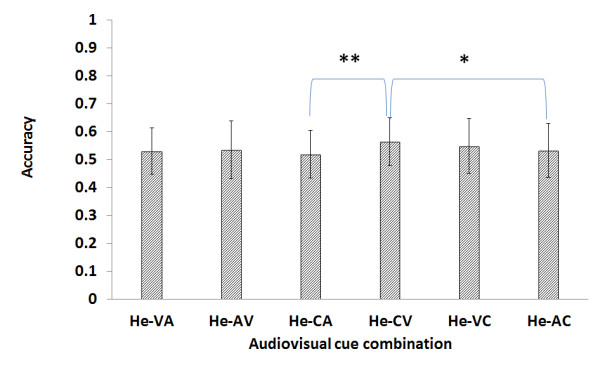
**Result of the naive heterogeneous experiment**. The He-CV (i.e., heterogeneous CV combination) accuracy is higher than the He-CA accuracy (p < 0.01) while the He-CV showed higher accuracy than He-AC (p < 0.05).

Two of these are specified with different cueing speeds, from which the term heterogeneity applies. Figure 5 shows the accuracy results of the six combinations of audio-visual cues described in Table 1. He-CV shows higher accuracy than He-CA (*p *< 0.01), and He-CV shows higher accuracy than He-AC (*p *< 0.05).

**Figure 6:**
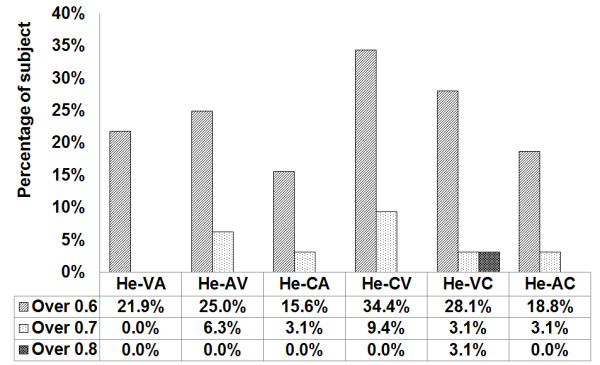
**Result of the naive heterogeneous experiment greater than 0.6 in terms of accuracy**. The experiment using heterogeneous training data shows the percentages of subject who scored greater than 0.6. He-CV and He-VC show higher percentage than others. He-CV shows higher percentage than He-VC.

Figure 6 shows the result of the analysis of the subjects whose average accuracy is greater than 0.6, 0.7, and 0.8 in terms of accuracy according to the heterogeneous cue combination of the He-CV combination of a combined cue for training and a visual cue for testing, and the He-VC combination of a visual cue for training and a combined cue for testing. He-CV and He-VC show higher percentage than others. He-CV shows higher percentage than He-VC.

The analysis criteria are based on the six combinations described in Table 1. The results shown in Figure 5 are more general with respect to the fact that they only show the general tendency in terms of the average. In comparison, Figure 6 is more indicative of the actual performance capacity, as this experiment included only subjects who scored above a certain level of accuracy.

## Discussion

### Experienced homogeneous data

The experienced homogeneous data is cross-compared against 3 classifiers, LS, SVM, and LDA. For the 10 subjects, the CV combination (training on a combined cue with testing was on a visual cue) showed the highest average accuracy. The same types of results were observed in all three of the classifiers (LS, SVM, and LDA). Furthermore, the combinations CA and CV out of the total of six showed the highest level of statistical significance (*p *< 0.05). On the basis of this result, an experiment was carried out on the 32 naive subjects. In this experiment, the comparison was made between experienced subjects using a combined cue as homogeneous training data and naive subjects using a combined cue as homogeneous training data.

### Naive homogeneous data

Out of a total of six combinations in the naive homogeneous training data, the CV combination showed the highest average accuracy, at 0.562. The second highest was the VC combinations, at 0.56. However, when the comparison is confined to the subjects whose average accuracy is greater than 0.6, the percentage of the VC combination is higher than that of CV, at 37.5% and 34.4%, respectively. It is particularly of note that the VC combination included two subjects whose average accuracy score was greater than 0.8. This shows that the average accuracy alone does not sufficiently explain the individual characteristics of the subjects with all six combinations. The CV combination used a combined cue for the training data and a visual cue for the test data, while the VC used a visual cue for the training data and a combined cue for the test data. In other words, the combination of CV and VC is a cross-combination of combined and visual cues. The results suggest that a combination of these two types of cues results in higher average accuracy.

### Naive heterogeneous data

The six cue-combinations used in the heterogeneous naive training data experiment showed that the CV combination led to the highest level of accuracy, at 0.564. The second highest level resulted from the VC cue, at 0.549. For the subjects with accuracy scores of 0.6 or above, the CV combination accounted for the highest percentage, at 34.4% and the second highest was VC at 28.1%. This shows a different trend from the homogeneous training data experiment.

### Cross-comparison between the homogenous and heterogeneous experiment

**Figure 7:**
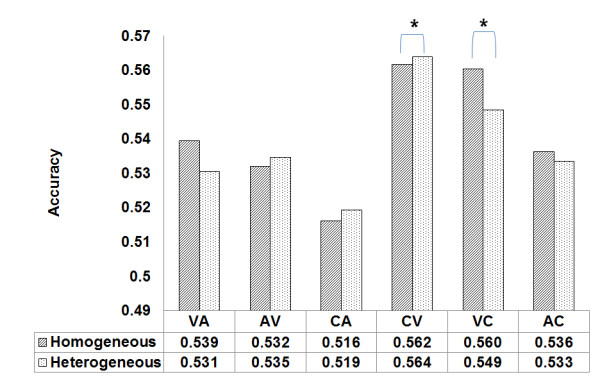
**Result of the comparison between homogeneous and heterogeneous experiment**. The heterogeneous CV combination is higher than the homogeneous CV combination (p < 0.05). For the VC combination, the heterogeneous accuracy is lower than the homogeneous accuracy (p < 0.05).

A cross-comparison between the homogeneous and heterogeneous experiments is carried out in this section to identify the factors that cause the aforementioned differences. Figure 7 shows comparisons of the average accuracy levels between the homogeneous and heterogeneous experiments on the basis of the six combinations described in Table 2.

On average, CV had the highest scores for both the homogeneous and heterogeneous criteria, and the heterogeneous data showed a higher average level of accuracy. In the case of VC, a contradictory result showed that the homogeneous data showed higher average accuracy levels than the heterogeneous data.

CV was trained on an audio-visual cue and tested on a visual cue. VC was trained on a visual cue and tested on an audio-visual cue. In both cases, there are common conditions that are designed to take advantage of an audio-visual cue and a visual cue as training or test data. In the end, the relationship between the two stimuli had a direct impact on the average performance. In the CV case, there was an improvement in the average performance in the experiment using the heterogeneous training data (*p *< 0.05), whereas in the VC case, there was decline in the average performance during the experiment using the heterogeneous training data (*p *< 0.05).

**Figure 8:**
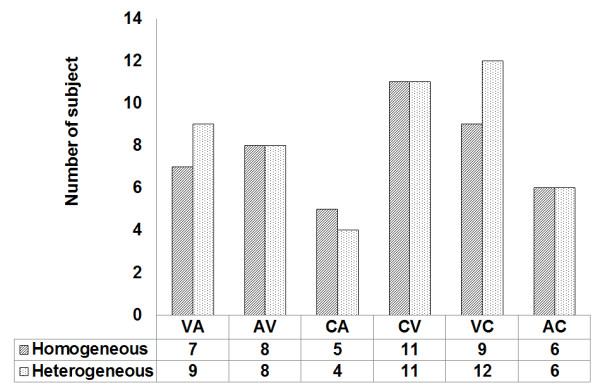
**Result of the comparison between the numbers of subjects who scored more than 0.6 in terms of accuracy**. CV and VC show higher results than the others. CV shows no changes between the homogeneous and heterogeneous stimuli but VC shows significant difference.

Figure 8 shows the comparisons between homogeneous and heterogeneous training data of the six cue-combinations, including the subjects whose average accuracy score is above 0.6. The CV case, whose training was on an audio-visual cue and whose testing was on a visual cue, showed no difference between the heterogeneous and homogeneous results. This suggests that the effect of the heterogeneity resulting from the cueing speed is minimal. On the other hand, the VC case showed a relatively significant effect in this regard.

The VC case, whose training was on a visual cue and whose performance was evaluated on an audio-visual cue, showed the highest percentage of subjects whose average accuracy score was above 0.6 in the homogeneous training experiment. However, this case showed a lower percentage than the CV case in the heterogeneous training experiment. This suggests that the VC case, whose training was on visual cue and whose testing was on an audio-visual cue, is not affected by changes in the cueing conditions. This is indicative of more consistent performance.

## Conclusions

When EEG-BCI-based motor imagery training tasks are carried out using naive subjects, the general applicability, stability and consistency of the accuracy levels are regarded the most essential. The set of experiments conducted here concluded that consistent accuracy can be achieved when the training data relies on a heterogeneous combined cue. Randomness of presenting time of the heterogeneous cue raises the power of concentration of the subjects, and this is thought to be the main cause of the consistency in performance.

An accuracy of the difference was 0.002 (*p *< 0.05) between 0.562 in the homogeneous one and 0.564 in heterogeneous one. A consistent result was obtained when the training data used a combined cue and the test data used a visual cue. Moreover, the combinations of a combined cue and a visual cue showed the highest at 0.562, 0.564 and the second highest accuracy at 0.56, 0.549.

We propose the use of this measuring methodology of a heterogeneous combined cue for training data and a visual cue as a testing cue by the typical EEG-BCI algorithm on the EEG-BCI system to achieve effectiveness in terms of consistency, stability, cost, time, and resources management without the need for a trial and error process.

## Competing interests

The authors declare that they have no competing interests.

## Authors' contributions

DC carried out the algorithm implementation and evaluation and drafted the manuscript. YR and YL conceived of the study and participated in its coordination. Both authors participated in the study design and in the data acquisition processes and both read and approved the final manuscript.
